# The Role of MALAT1 in Regulating the Proangiogenic Functions, Invasion, and Migration of Trophoblasts in Selective Fetal Growth Restriction

**DOI:** 10.3390/biom14080988

**Published:** 2024-08-11

**Authors:** Shuting Xia, Yingnan Ye, Jialiu Liu, Hanfei Qiu, Minhuan Lin, Zhiming He, Linhuan Huang, Malie Wang, Yanmin Luo

**Affiliations:** 1 Department of Obstetrics & Gynecology, The First Affiliated Hospital of Sun Yat-Sen University, Guangzhou 510080, China; xiasht3@mail2.sysu.edu.cn (S.X.); yeyn3@mail3.sysu.edu.cn (Y.Y.); liujliu@mail2.sysu.edu.cn (J.L.); qiuhf3@mail2.sysu.edu.cn (H.Q.); linmh26@mail.sysu.edu.cn (M.L.); hezhim5@mail.sysu.edu.cn (Z.H.); hlhuan@mail.sysu.edu.cn (L.H.); wangmal@mail.sysu.edu.cn (M.W.); 2 Guangdong Provincial Clinical Research Center for Obstetrical and Gynecological Diseases, Guangzhou 510080, China

**Keywords:** selective fetal growth restriction, monochorionic twins, placenta, trophoblast, endothelial angiogenesis, paracrine, metastasis-associated lung adenocarcinoma transcript 1

## Abstract

Epigenetic regulation is an important entry point to study the pathogenesis of selective fetal growth restriction (sFGR), and an understanding of the role of long noncoding RNAs (lncRNAs) in sFGR is lacking. Our study aimed to investigate the potential role of a lncRNA, metastasis-associated lung adenocarcinoma transcript 1 (MALAT1), in sFGR using molecular biology experiments and gain- or loss-of-function assays. We found that the levels of MALAT1, ERRγ, and HSD17B1 were downregulated and that of miR-424 was upregulated in the placental shares of the smaller twins. Moreover, angiogenesis was impaired in the placental share of the smaller fetus and MALAT1 could regulate the paracrine effects of trophoblasts on endothelium angiogenesis and proliferation by regulating miR-424. In trophoblasts, MALAT1 could competitively bind to miR-424 to regulate the expression of ERRγ and HSD17B1, thus regulating trophoblast invasion and migration. MALAT1 overexpression could decrease apoptosis and promote proliferation, alleviating cell damage induced by hypoxia. Taken together, the downregulation of MALAT1 can reduce the expression of ERRγ and HSD17B1 by competitively binding to miR-424, impairing the proangiogenic effect of trophoblasts, trophoblast invasion and migration, and the ability of trophoblasts to compensate for hypoxia, which may be involved in the pathogenesis of sFGR through various aspects.

## 1. Introduction

Twins accounted for 1.5–3% of all births in the USA and the UK in last decade [1], and a similar birth rate of twins of around 3% was also found in China [2], of which 20–30% are monochorionic (MC) twin pregnancies [3]. MC twins are prone to some unique complications, like selective fetal growth restriction (sFGR), twin-to-twin transfusion syndrome (TTTS), etc., resulting in increased perinatal adverse outcomes [4]. sFGR affects 10–15% of MC twin pregnancies, with an increased risk of intrauterine fetal death, severe premature delivery, and nervous system developmental disorders in both the small-for-gestational-age and the appropriate-for-gestational-age twins, which is one of the serious complications that may occur in MC twins [5].

Previous studies have suggested that the occurrence of sFGR is related to placental dysfunction in the smaller twin caused by an uneven placental share, placental malperfusion, and hypoxic stress, etc., involving multiple pathophysiological processes, including impaired placental angiogenesis, trophoblast dysfunction, and hypoxia-induced trophoblast damage [6,7], but its specific molecular mechanism remains unclear. Generally, sFGR twins harbor an almost identical genetic background and develop in the same intrauterine environment, so epigenetic regulatory mechanisms of non-DNA sequence changes, such as DNA methylation and micro RNAs (miRNAs) that regulate placental development and function at the transcriptional or post-transcriptional levels [8,9] may be an important entry point to study the pathogenesis of sFGR. Our previous study found that the increased miR-424 in the placenta of fetal growth restriction inhibited the expression of the transcription factor estrogen related receptor gamma (ERRγ) and its downstream gene 17 beta-hydroxysteroid dehydrogenase type 1 (HSD17B1) and impaired the proliferation and invasion of trophoblasts [10]. Using database prediction [11], we found that a long non-coding RNA, metastasis-associated lung adenocarcinoma transcript 1 (MALAT1), may act as a molecular sponge of miR-424 to regulate the expression of its downstream genes.

Long non-coding RNAs (lncRNAs), defined as RNAs longer than 200 nucleotides without or with the limited capacity of coding proteins, are also an important part of epigenetics research, and accumulating evidence has shown that aberrant lncRNA expression is associated with various pregnancy complications [12,13]. MALAT1 is a highly conserved lncRNA in mammals, which is often regulated by stress, such as hypoxia, hyperglycemia, infection, etc., and then regulates cell biological functions, like proliferation, apoptosis, and angiogenesis [14,15]. It has also been studied in pregnancy-related diseases, mainly in preeclampsia [16,17], but its relationship with fetal growth is under elucidated. Since MC twins are fundamentally monozygotic, MC twins with discordant phenotypes are considered an optimal model for investigating epigenetic molecular mechanisms by using co-twins as internal controls [18,19]. Thus, we sought to study the regulatory role of MALAT1 in fetal growth in sFGR to minimize the effect of differences in genetic backgrounds among individuals. In the current study, using gain- or loss-of-function experiments, we validated that decreased MALAT1 would impair the paracrine effect of trophoblasts on endothelial cell and trophoblast biological functions through the miR-424/ERRγ/HSD17B1 axis and meanwhile decrease trophoblast survival under hypoxia, suggesting that MALAT1 is involved in the pathogenesis of sFGR through multiple aspects.

## 2. Materials and Methods

### 2.1. Participants and Placental Tissue Collection

This study was approved by the Medical Ethics Committee of the First Affiliated Hospital of Sun Yat-Sen University (Application ID: [2021]210-2), and informed consent was signed by all participants before sample collection. Monochorionic diamniotic (MCDA) twin pregnancies diagnosed with or without sFGR were enrolled. MCDA was determined via prenatal ultrasound in the first trimester and confirmed based on the postnatal placenta examination with only one placenta and 2 layers of amniotic membranes between two fetuses. sFGR was defined as the estimated fetal weight (EFW) of one twin < 3rd centile or satisfying at least two out of four parameters (EFW of one twin < 10th centile, abdominal circumference of one twin < 10th centile, intertwin EFW discordance > 25%, and umbilical artery pulsatility index of the smaller twin > 95th centile) [20]. The intertwin EFW discordance was calculated as follows: [(EFW of the larger twin − EFW of the smaller twin)/EFW of the larger twin]. The exclusion criteria for samples were as follows: pregnancies complicated by intrauterine infection, intrauterine fetal death, twin-to-twin transfusion syndrome (TTTS), twin anemia polythaemia sequence (TAPS), twin reversed arterial perfusion (TRAP), congenital anomalies, and birthweights of both twins below the 10th percentile of the gestation. All cases were delivered via cesarean section in the third trimester. Within 30 min after delivery, the placenta was immediately examined to confirm the chorionicity, and the territories of the twins were identified according to their vascular distribution. After removing the chorion and decidua, three fresh samples were collected randomly away from cord insertion and at least 2 cm far apart from each other from the territories of each of the twins and rinsed three times in ice-cold saline (0.9% NaCl) to remove excessive maternal blood. The samples from the sFGR twin and its larger co-twin of the sFGR twin pairs were denoted as sFGR-S and sFGR-L, respectively, while the samples from the relatively smaller and the relatively larger twins of the control normal twin pairs were denoted as control-S and control -L, respectively. All placental specimens were further divided into 3 parts and stored separately in various preparations for different analyses. For total RNA isolation, the placental tissues were immediately immersed in RNAlater (Thermo Fisher Scientific, Waltham, MA, USA), stored at 4 °C overnight, and kept in −80 °C until further experiments. For protein extraction, the placental tissues were snap-frozen in liquid nitrogen and stored at −80 °C. For immunofluorescent and immunohistochemistry staining, the remaining placental tissues were further dissected into a size of about 1 cm*1 cm*1 cm and fixed in 4% paraformaldehyde. Then, the placental samples were embedded in a random orientation in paraffin.

### 2.2. Cell Culture

The human extravillous trophoblast cell line HTR-8/SVneo was purchased from the American Type Culture Collection (ATCC, Manassas, VA, USA) and cultured in RPMI 1640 medium (Gibco, Carlsbad, CA, USA) supplemented with 10% fetal bovine serum (FBS, Gibco) and 1% penicillin/streptomycin (Gibco), in humidified air at 37 °C with 5% CO_2_. Hypoxic culturing was performed by incubating HTR-8/SVneo cells in the anaerobic/microaerobic culture system (MARK III AN3, Anoxomat, Advanced Instruments, Bedford, MA, USA) at an oxygen concentration of 2%.

Primary HUVECs were isolated from the freshly obtained human umbilical cords of healthy singleton pregnancies without complications, and informed consent was obtained. Briefly, the cord was perfused with 0.25% trypsin-EDTA (Gibco, Carlsbad, CA, USA) and digested for 7 min and 12 min at 37 °C separately. Then, the solution containing HUVECs was flushed from the cord via perfusion with ECM (ScienCell, Carlsbad, CA, USA) supplemented with 10% FBS to stop digestion. After centrifugation at 1000 rpm for 5 min, cells were resuspended in ECM containing 10% FBS and 1% penicillin/streptomycin. The HUVECs were placed in a culture flask and incubated in humidified air at 37 °C with 5% CO_2_.

### 2.3. RNA Extraction, Reverse Transcription, and Real-Time Quantitative PCR (RT-qPCR)

Total RNA, including miRNAs, was extracted from placental tissues or cultured cells using an miRNeasy Tissue/Cells Advanced Mini Kit (217604, QIAGEN, Venlo, The Netherlands) following the manufacturer’s instructions. Equal amounts of RNA were subjected to cDNA synthesis (A0010CGQ and EZB-miRT2, EZBioscience, Roseville, MN, USA), and qRT-PCR was performed using SYBR Green Master Mix (A0012-R2, EZBioscience) on a CFX96 Real-Time PCR System (Bio-Rad, Hercules, CA, USA). U6 and β-actin were used as the internal controls to normalize the miRNA and mRNA expression levels, respectively, in each sample, and the relative gene expression levels were calculated with the 2^−ΔΔCt^ method. The sequences of the specific primers are shown in Table 1.

### 2.4. Western Blotting

The total proteins were extracted from placental tissues or HTR-8/SVneo cells using RIPA lysis buffer (P0013B, Beyotime, Shanghai, China) and quantified with a bicinchoninic acid (BCA) protein assay kit (CW0014S, CWBIO, Shanghai, China). The proteins were incubated for 10 min at 100 °C, and then, equal amounts of protein were loaded onto 10% SDS-PAGE gels and transferred to polyvinylidene difluoride (PVDF) membranes (0.22 mm pore size; Millipore, Burlington, MA, USA). The blots were incubated with 5% skim milk in Tris-buffered saline containing 0.05% Tween 20 (TBST) at room temperature for 1 h and then incubated overnight at 4 °C with the rabbit monoclonal anti-ERRγ (1:1000 for cell lysates and 1:500 for tissue lysates, ab128930, Abcam, Cambridge, UK), rabbit monoclonal anti-HSD17B1 (1:1000 for cell lysates and 1:10,000 for tissue lysates, ab51045, Abcam), or anti-beta tubulin (1:5000, 10068, Proteintech, Rosemont, IL, USA) primary antibodies. Subsequently, the membranes were washed with TBST and incubated with secondary antibodies (1:5000, Proteintech) for 1 h at room temperature. The proteins were visualized using the chemiluminescence method (WBKLS0500, Millipore), according to the manufacturer’s recommendations, and the relative gray value of the protein bands was calculated by using beta tubulin as the internal control.

### 2.5. Fluorescence In Situ Hybridization (FISH)

The FISH assay was performed on paraffin-embedded placental tissues. Double-digoxigenin–labeled (DIG-labeled), MALAT1-specific probes were designed and synthesized by Umine Biotechnology Co., LTD (Guangzhou, China). Briefly, after deparaffinization and rehydration, the samples were digested with proteinase K (Sangon Biotech, Shanghai, China) for 30 min at 37 °C and subsequently dehydrated. The sections were then hybridized with the probe overnight at 37 °C in hybridization solution. After stringent washes, the sections were incubated at room temperature (RT) for 1 h with an anti-DIG-horseradish peroxidase (HRP) antibody (1:200, Focobio, Guangzhou, China). Then, the sections were stained with tyramide signal amplification (TSA) solution, counterstained with 4′,6-diamidino-2-phenylindole (DAPI, Beyotime, Jiangsu, China), and mounted. Images were obtained using a fluorescence microscope (Pannoramic MIDI, 3DHISTECH, Budapest, Hungary).

### 2.6. Immunohistochemistry (IHC) Staining and Analysis of Angiogenesis

The procedures for IHC detection are described below. First, for antigen retrieval, rehydrated paraffin sections were heated in citrate buffer (pH 6.0) for 15 min. Then, endogenous peroxidase activity was quenched by incubating the sections with 3% H_2_O_2_ for 30 min and blocking the sections with 10% normal goat serum for 30 min at room temperature. After washing, sections were incubated overnight at 4 °C with an anti-CD31 (1:2000, ab182981, Abcam, Cambridge, UK) primary antibody, as a marker of angiogenesis. Finally, the slides were incubated with HRP-conjugated secondary anti-rabbit immunoglobulin G (1:2000, ab205718, Abcam) at RT for 45 min. Diaminobenzidine tetrahydrochloride (DAB) was used as a substrate, and sections were lightly counterstained with hematoxylin, dehydrated, and mounted. All slides were examined under an optical microscope (NanoZoomer S360, HAMAMATSU PHOTONICS, Shizuoka, Japan) with 100× magnification. Placental angiogenesis was assessed based on CD31-positive fetal capillary counts and the volume density of vessels in villi as previously reported [9]. Specifically, 5 micrographs for each slide were captured and a 99-point square grid was applied to each micrograph (total number of test points = 99). Points hitting CD31-positive endothelial cells were counted, and the volume density was determined based on the number of hitting points/total number of test points × 100%.

### 2.7. Cell Transfection

Small interfering RNA (siRNA) targeting MALAT1 (siMALAT1) and a scrambled siRNA used as a negative control (scramble) were purchased from Umine Biotechnology Co., Ltd. (Guangzhou, China). The GV712 vector (Genechem, Shanghai, China) was used for MALAT1 overexpression (oeMALAT1), and an empty plasmid vector was used as a negative control (vector). The inhibition and overexpression of miR-424-5p were achieved using a miR-424 inhibitor and miR-424 mimic, respectively (GenePharma Co., Ltd., Suzhou, China). These oligonucleotides and plasmids were transfected into cells using Lipofectamine 3000 Reagent (Thermo Fisher Scientific, Waltham, MA, USA) according to the manual. The cells were transfected for 48 h or 72 h and then collected for subsequent analyses.

### 2.8. Preparation of Conditioned Medium (CM) of Trophoblasts

At 24 h after transfection of the corresponding molecules into HTR-8/SVneo cells, cells of each group were digested, counted, and plated in 12-well plates at a number of 300,000 cells per well. After another 24 h, the medium was replaced with 1 mL RPMI 1640 without FBS. After 48 h of continuous culture, the cell culture supernatant of each group was collected, centrifuged at the speed of 1000 rpm for 5 min, filtered with a sterile syringe filter with a 0.22 µm pore size (Millipore, Burlington, MA, USA), and then kept in −80 °C immediately.

### 2.9. Tube Formation Assay

Matrigel (BD Biosciences, Franklin Lakes, NJ, USA) was melted at 4 °C, and the 96-well plates and pipette tips needed were precooled at −20 °C before the experiment. Matrigel was added at 80 μL per well, being careful not to have bubbles, and then incubated at 37 °C for 1 h. After co-culture with trophoblast CM for 48 h, each group of endothelial cells was digested and counted, and 20,000 cells were plated on the Matrigel in 100 μL FBS-free ECM. After incubation at 37 °C and 5% CO_2_ for 6 h, photographs were taken under an inverted microscope (DMI1, Leica), and ImageJ software (Version 1.53v) was used for analysis.

### 2.10. Immunofluorescent (IF) Staining of Ki-67

Ki-67 is a nuclear protein antigen characterized by its absence in resting cells and expression in proliferating cells, so it is commonly used as a marker for cell proliferation by detecting and analyzing Ki-67 through immunohistochemical staining or other techniques [21]. In our present study, we compared differences in endothelial cell proliferation in different CMs of trophoblasts via IF staining for Ki-67. Briefly, endothelial cells were digested, counted, and plated in 96-well plates at a number of 6000 per well and co-cultured with trophoblast CM for 48 h. After washing gently 3 times with PBS, cells were fixed in 4% paraformaldehyde at room temperature for 15 min, treated with 0.2% Triton X-100 at room temperature for 10 min, and blocked with 5% BSA for 30 min. Then, cells were incubated overnight at 4 °C with the rabbit monoclonal anti-Ki-67 antibody (1:500, ab128930, Abcam, Cambridge, UK). Subsequently, cells were incubated with a Cy3-goat anti rabbit secondary antibody (1:200, Beyotime, Jiangsu, China) for 1 h at room temperature and DAPI for 10 min. Fluorescent images were taken under an inverted fluorescent microscope (DMI8, Leica), and the rates of Ki-67-positive cells were calculated through the use of ImageJ software.

### 2.11. Dual-Luciferase Reporter Assay

The pmirGLO plasmids containing the wild-type (WT) 3’-untranslated region (3’-UTR) or mutant (MUT) 3’-UTR of MALAT1 were purchased from Umine Biotechnology Co., Ltd. (Guangzhou, China). The recombinant luciferase reporter plasmids MALAT1-WT or MALAT1-MUT were co-transfected into HTR-8/SVneo cells with the mimic NC or miR-424-5p mimic, respectively. At 48 h after transfection, the cell lysates were collected after centrifugation at the speed of 12,000× *g* for 5 min. The Dual Luciferase Reporter Gene Assay Kit (RG027, Beyotime, Shanghai, China) was used to determine the relative luciferase units (RLUs), with firefly luciferase serving as the internal reference.

### 2.12. Cell Proliferation Assay

The cell proliferation assay was performed using a CCK-8 assay (APExBIO, Houston, TX, USA). Cells were seeded on 96-well plates at a density of 6000 cells/100 μL per well. The proliferation level was determined at 0, 24, 48, and 72 h after the transfection of oligonucleotides or plasmids; 10 μL of CCK-8 solution was added to each well, followed by 2 h of incubation in the dark at 37 °C. The absorbance at 450 nm was measured using a microplate reader (Infinite F50, Tecan, Männedorf, Switzerland).

### 2.13. Transwell Invasion Assay

A total of 200 µL of cells suspended in serum-free medium (1.25 × 10^5^ cells/mL) was seeded into the upper chamber of cell culture inserts (24-well, 8 μm pore size; BD Biosciences, Franklin Lakes, NJ, USA) precoated with a Matrigel (BD Biosciences) membrane. A total of 600 µL of RPMI 1640 medium containing 10% FBS was added to the lower chamber as a chemoattractant. After 24 h of incubation at 37 °C, the chamber was removed and gently washed twice with PBS followed by fixation with 4% paraformaldehyde for 15 min. Then, the cells that had invaded across the membrane were stained with crystal violet for 15 min at room temperature. The numbers of invasive cells were observed under an inverted microscope (DMI1, Leica) and counted and averaged from five randomly selected fields.

### 2.14. Wound Healing Assay

The wound healing assay was performed to assess the cell migration capacity. In brief, cells, after the transfection of oligonucleotides or plasmids, were cultured to a confluence of 85–90%. A wound of approximately 2 mm in width was made by manually scraping the monolayer in the middle of each well with a 200 µL pipette tip. The wound was photographed under an inverted microscope (DMI1, Leica Microsystems, Bannockburn, IL, USA) at 0 and 12 h after scratching. ImageJ software was used to calculate the cell-free area at each time point, which was compared with that at the 0 h time point to calculate the wound closure (migration rate).

### 2.15. Apoptosis Detected via Flow Cytometry

The supernatant of the cell culture and digested cells was collected together, washed with pre-cooled 2 times, and then resuspended in 1× Binding Buffer at a concentration of 1 × 10^6^/mL. Cells were incubated with 5 μL of Annexin V-FITC and PI, respectively, at room temperature for 15 min in the dark. Finally, another 400 μL of 1× Binding Buffer was added to each tube, and the results were recorded via flow cytometry (Cytoflex, BD Biosciences).

### 2.16. Statistical Analysis

Data of clinical cases were expressed as the median (interquartile range), and experimental data were expressed as the mean ± standard deviation or percentage relative to the control group. ImageJ software was used for the analysis of images of the wound healing and transwell invasion assay, and GraphPad Prism 9.0 (GraphPad Software, Boston, MA, USA) was used for statistical analyses. The Mann–Whitney test or Student’s t test was used to compare two independent samples. One-way ANOVA and the least significant difference (LSD) multiple comparison tests were used to compare the differences among groups. *p* < 0.05 indicated statistical significance. All data were obtained from at least 3 independent experiments.

## 3. Results

### 3.1. Participant Demographics

We first measured the relative expression of MALAT1, ERRγ, HSD17B1, and miR-424 in placental tissues obtained from the sFGR group (n = 8) and control group (n = 8). Finally, eight sFGR (sFGR group) and eight MC twin pregnancies with intertwin EFW discordance < 25% (control group) were included. The clinical characteristics of the two groups are listed in the Table 2. The birth weights of both the smaller and larger fetuses were significantly lower in the sFGR group than in the control group, and the birth weight discordance was significantly greater in the sFGR group than in the control group (*p* < 0.001). The placental share of the smaller sFGR twins (22.2%) was significantly less than that of the smaller twins in the control group (41.7%) (*p* < 0.001).

### 3.2. Decreased Expression of MALAT1, ERRγ, and HSD17B1 in the Placental Share of the Smaller Fetus in sFGR Pregnancies

**Figure 1:**
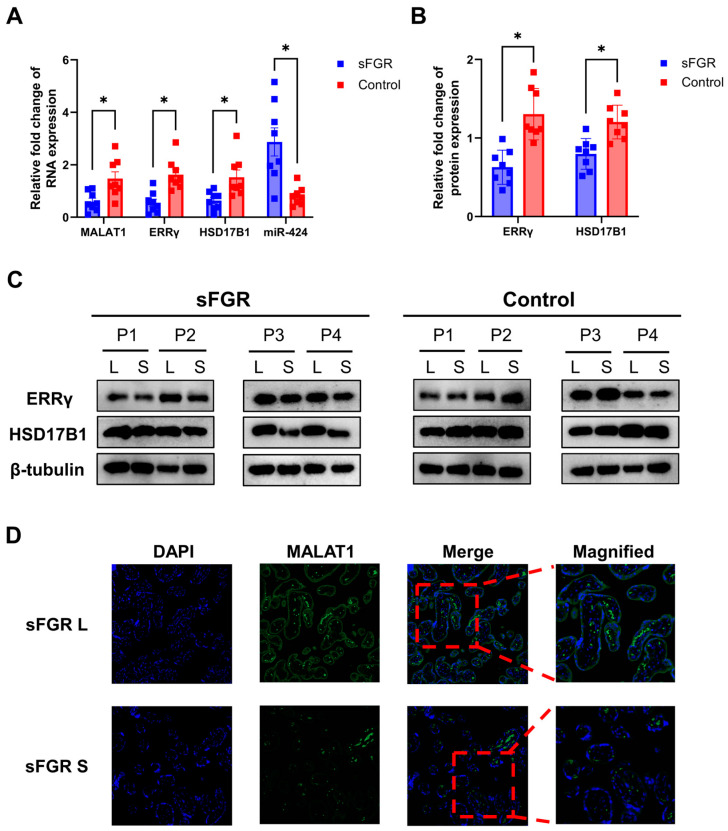
MALAT1, ERRγ, and HSD17B1 expression was decreased and miR-424 was increased in the placental share of the smaller fetus in sFGR pregnancies. (**A**) RT-qPCR results the showing relative fold-change in RNA expression levels of corresponding molecules in the placental share of the smaller fetus compared to those of the larger fetus in the sFGR (n = 8) and control (n = 8) groups. (**B**) The quantitative analysis of the relative fold-change in protein expression levels of ERRγ and HSD17B1 in the placental share of the smaller fetus compared to those of the larger fetus in sFGR (n = 8) and control (n = 8) groups, assessed via Western blotting, and representative images of blots are shown in (**C**). * *p* < 0.05 vs. control. (**D**) Representative FISH images of placental regions in sFGR (×100 for the left three panels, ×200 for the right magnified fields). Statistical data were measurement data and described as the mean ± standard deviation. L, larger twins; S, smaller twins. Original western blot and fluorescent images can be found in the Supplementary Materials.

In the sFGR group, MALAT1, ERRγ, and HSD17B1 mRNA levels were significantly decreased in the placental shares of the smaller twins compared with the larger twins (Supplementary Figure S1A). The average fold-changes of MALAT1, ERRγ, and HSD17B1 mRNA levels in the smaller twins compared to the larger twins were 0.60, 0.55, and 0.64, respectively, which were significantly reduced compared to the control group (1.47, 1.62, and 1.52, respectively; Figure 1A and Supplementary Figure S1C). Conversely, the average fold-change of miR-424 expression in the placental shares of the smaller twins was 2.87 relative to the larger twins in the sFGR group, significantly higher than that in the control group (0.81-fold). There was no significant difference in MALAT1, ERRγ, HSD17B1, and miR-424 expression between larger twins in the sFGR group and twins in the control group (Supplementary Figure S1E). Similarly, results showed that protein levels of ERRγ and HSD17B1 were decreased in the placental shares of the smaller twins compared with the larger twins in sFGR pregnancies but were comparable between twins in the control group (Figure 1B,C, Supplementary Figure S1B,D,F). Then, the fluorescence in situ hybridization (FISH) assay showed that MALAT1 mainly localized to the trophoblast cytoplasm and was downregulated in placental tissues from the smaller fetus compared with the larger fetus in sFGR pregnancies (Figure 1D).

### 3.3. Angiogenesis Is Impaired in the Placental Share of the Smaller Fetus in sFGR Pregnancies and Conditioned Medium of MALAT1-Decreased Trophoblasts

**Figure 2:**
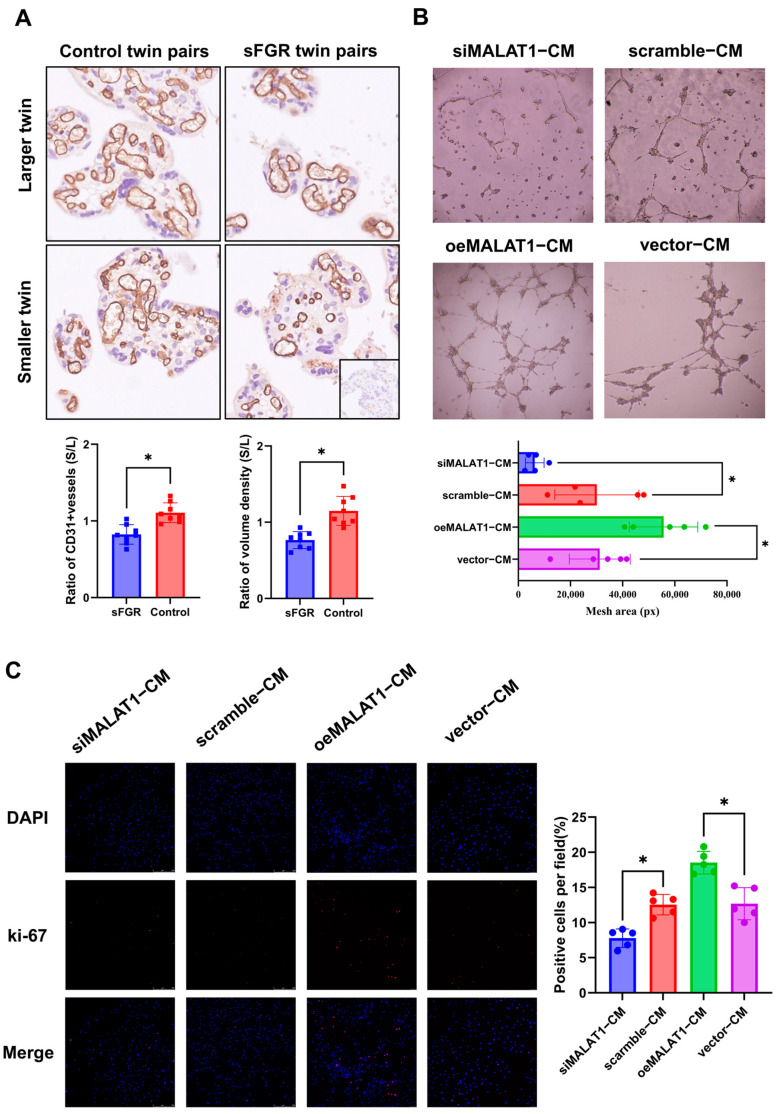
Angiogenesis was impaired in the placental share of the smaller fetus in sFGR pregnancies, which may be associated with decreased MALAT1 (**A**) Representative images of CD31 immunohistochemical staining (×200) of placental regions in the sFGR and control group, and quantitative analysis of CD31-positive vessel counts and volume density (n = 8 for each group). (**B**) Representative images of HUVEC tube formation assays after co-culture with CM from trophoblasts transfected with siMALAT1, negative controls (scramble), oeMALAT1, or negative controls (vector). (**C**) Representative images of Ki-67 immunofluorescent staining (×100) after co-culture with CM from trophoblasts transfected with siMALAT1, negative controls (scramble), oeMALAT1, or negative controls (vector). * *p* < 0.05. Statistical data were measurement data and described as the mean ± standard deviation. Original images can be found in the Supplementary Materials.

The ratio of CD31-positive fetal capillary counts and volume density within villi in the placental share of the smaller fetus to that in the larger fetus in sFGR twin pairs was significantly lower when compared to the ratio in the control twin pairs (0.82 vs. 1.10 and 0.76 vs. 1.15, *p* < 0.05, Figure 2A), indicating that the placental angiogenesis was impaired in sFGR twin pregnancies.

Further, we decreased or increased MALAT1 expression in a commonly used trophoblastic cell line, HTR-8/SVneo cells, with MALAT1 siRNAs or plasmids, using scrambled siRNAs or empty vectors as corresponding negative controls. The culture supernatant of trophoblasts, namely the conditioned medium (CM), under different transfections was collected respectively. The effects of different CMs on HUVEC angiogenesis and proliferation were analyzed in vitro. Results of the tube formation assay showed that CM from MALAT1-silenced trophoblasts significantly decreased, while CM from MALAT1-overexpressing trophoblasts significantly enhanced, the angiogenesis ability of HUVECs (Figure 2B). Immunofluorescence analysis of Ki-67-positive cells showed that the CM from MALAT1-silenced trophoblasts significantly decreased, while CM from MALAT1-overexpressing trophoblasts significantly enhanced, the proliferation ability of HUVECs (Figure 2C). Therefore, we found that MALAT1 can regulate the paracrine effects of HTR-8/SVneo cells on HUVEC angiogenesis and proliferation, which may contribute to the impaired angiogenesis in the placental share of the smaller fetus in sFGR pregnancies.

### 3.4. MALAT1 Serves as a Sponge of miR-424 to Regulate ERRγ and HSD17B1 Expression

**Figure 3:**
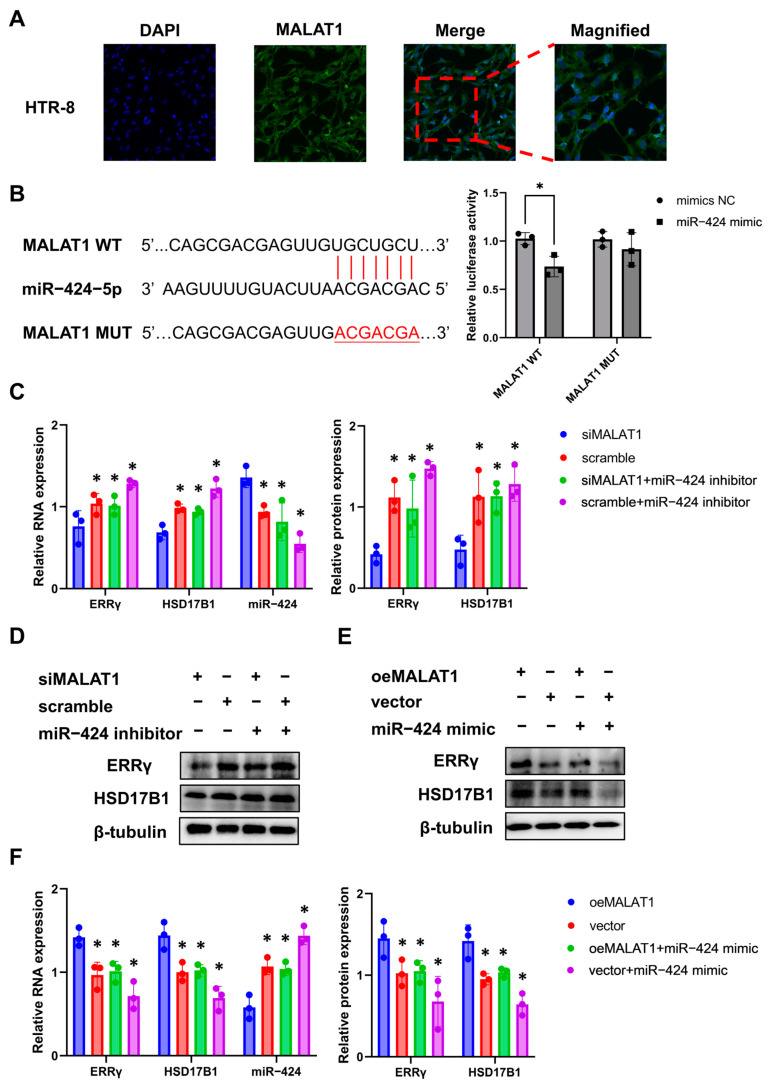
MALAT1 served as a sponge of miR-424 to regulate ERRγ and HSD17B1 expression. (**A**) The subcellular localization of MALAT1 in HTR-8/SVneo cells detected via FISH (×200 for the left three panels, ×400 for the right magnified fields). (**B**) The specific binding sites between miR-424 and MALAT1 were predicted and verified via a dual-luciferase reporter assay. * *p* < 0.05 vs. the mimic NC group. (**C**) The expression of corresponding molecules in response to the treatment of siMALAT1 and negative controls (scramble), with or without the miR-424 inhibitor, determined via RT-qPCR, and quantitative analysis of Western blot results of ERRγ and HSD17B1. * *p* < 0.05 vs. the siMALAT1 group. (**D**) The Western blot results of ERRγ and HSD17B1 in response to treatment with siMALAT1 and negative controls (scramble), with or without the miR-424 inhibitor. (**E**) The Western blot results of ERRγ and HSD17B1 in response to treatment with oeMALAT1 and negative controls (vector), with or without the miR-424 mimic. (**F**) The expression of corresponding molecules in response to treatment with oeMALAT1 and negative controls (vector), with or without the miR-424 mimic determined via RT-qPCR, and quantitative analysis of Western blot results of ERRγ and HSD17B1. * *p* < 0.05 vs. the oeMALAT1 group. Statistical data were measurement data and described as the mean ± standard deviation. Original western blot and fluorescent images can be found in the Supplementary Materials.

Then, we sought to investigate the mechanism through which MALAT1 regulates the paracrine effects of trophoblasts. As miR-424 was increased and ERRγ and HSD17B1 were decreased in the placental shares of the smaller twins in sFGR (Figure 1) and MALAT1-silenced trophoblasts (Supplementary Figure S2), we further explored the relationship between MALAT1 and the miR-424/ERRγ/HSD17B1 axis. Results of the FISH assay showed that MALAT1 was also located in the cytoplasm of HTR-8/SVneo cells (Figure 3A). Based on these findings, we speculated that MALAT1 might regulate ERRγ and HSD17B1 expression by sponging miR-424. Then, the binding site between miR-424 and MALAT1 was predicted using the RNA hybrid website and verified via a dual-luciferase reporter assay, which showed that the miR-424 mimic inhibited the luciferase activity of wild-type (WT) MALAT1 (*p* < 0.05), but that of the mutant (MUT) MALAT1 remained unaffected (Figure 3B).

Next, trophoblasts were transfected with only siMALAT1 or oeMALAT1 and co-transfected with siMALAT1 and the miR-424 inhibitor or oeMALAT1 and the miR-424 mimic, and the expression of miR-424, ERRγ, and HSD17B1 was measured via RT-qPCR and Western blotting. Results showed that MALAT1 knockdown significantly upregulated miR-424 expression and downregulated ERRγ and HSD17B1 expression, while co-transfection with the miR-424 inhibitor reversed the decreased expression of ERRγ and HSD17B1 (Figure 3C,D). MALAT1 overexpression regulated their expression reversely, and co-transfection with the miR-424 mimic decreased the enhanced expression of ERRγ and HSD17B1 in the oeMALAT1 group (Figure 3E,F). Taken together, decreased MALAT1 impaired the expression of ERRγ and HSD17B1 in trophoblasts by competitively binding to miR-424.

### 3.5. MALAT1 Regulates the Paracrine Effects of Trophoblasts on the Endothelium, Trophoblast Invasion, and Migration by Sponging miR-424

**Figure 4:**
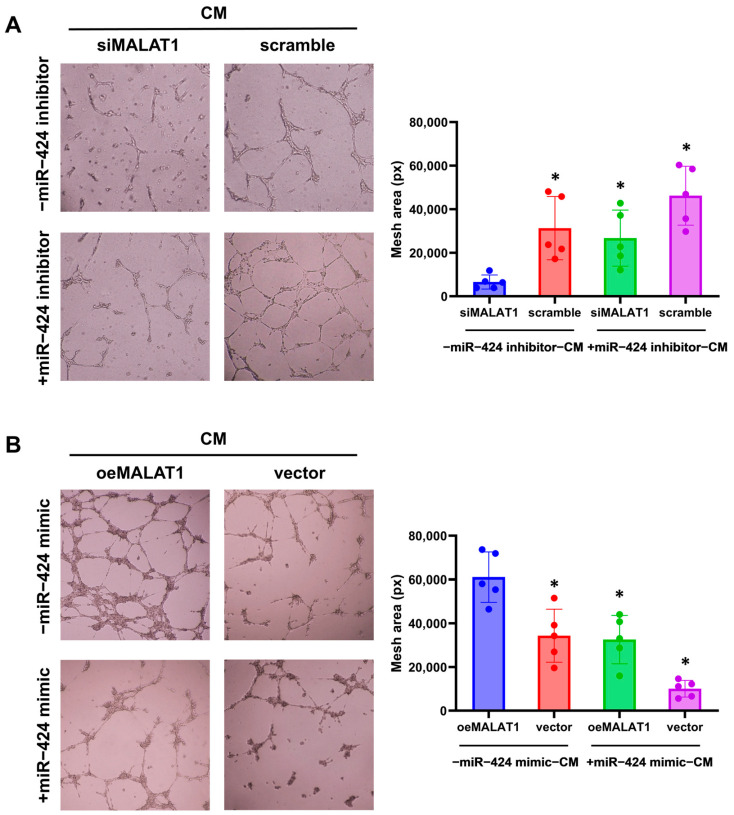
MALAT1 regulates the paracrine effects of trophoblasts on endothelium tube formation through miR-424. (**A**) Representative images of HUVEC tube formation assays after co-culture with CM from trophoblasts transfected with siMALAT1 and negative controls (scramble), with or without the miR-424 inhibitor and quantitative results. * *p* < 0.05 vs. the siMALAT1 group. (**B**) Representative images of HUVEC tube formation assays after co-culture with CM from trophoblasts transfected with oeMALAT1 and negative controls (vector), with or without the miR-424 mimic and quantitative results. * *p* < 0.05 vs. the oeMALAT1 group. Statistical data were measurement data and described as the mean ± standard deviation. Original images can be found in the Supplementary Materials.

**Figure 5:**
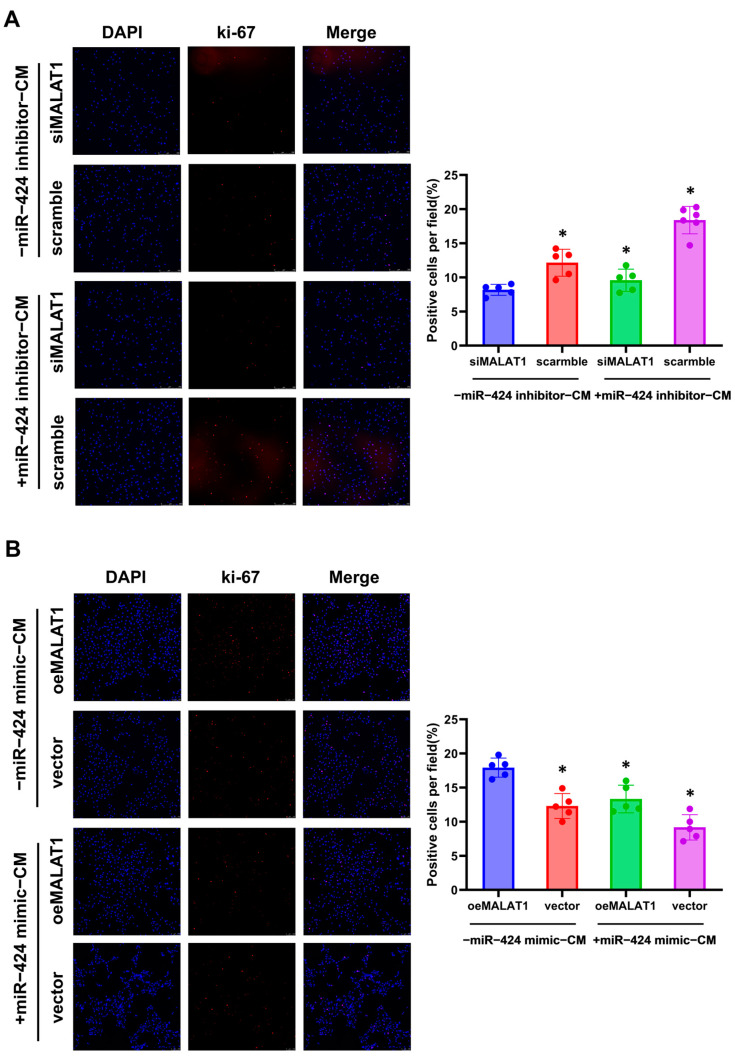
MALAT1 regulates the paracrine effects of trophoblasts on endothelium proliferation through miR-424. (**A**) Representative images of Ki-67 immunofluorescent staining (×100) after co-culture with CM from trophoblasts transfected with siMALAT1 and negative controls (scramble), with or without the miR-424 inhibitor and quantitative analysis of Ki-67-positive cells. * *p* < 0.05 vs. the siMALAT1 group. (**B**) Representative images of Ki-67 immunofluorescent staining (×100) after co-culture with CM from trophoblasts transfected with oeMALAT1 and negative controls (vector), with or without the miR-424 mimic and quantitative analysis of Ki-67-positive cells. * *p* < 0.05 vs. the oeMALAT1 group. Statistical data were measurement data and described as the mean ± standard deviation. Original images can be found in the Supplementary Materials.

We next investigated whether MALAT1 regulates the paracrine effects of trophoblasts by mediating miR-424. Results of the tube formation assay showed that miR-424 expression reversed the impaired angiogenic paracrine effect from MALAT1-silenced trophoblasts and the enhanced angiogenic paracrine effect from MALAT1-overexpressing trophoblasts significantly (Figure 4). Immunofluorescence analysis of Ki-67-positive cells showed that the decreased proliferation ability of HUVECs cultured with CM from MALAT1-silenced trophoblasts and the increased proliferation ability with CM from MALAT1-overexpressing trophoblasts were also significantly influenced by the change in miR-424 expression in trophoblasts (Figure 5).

**Figure 6:**
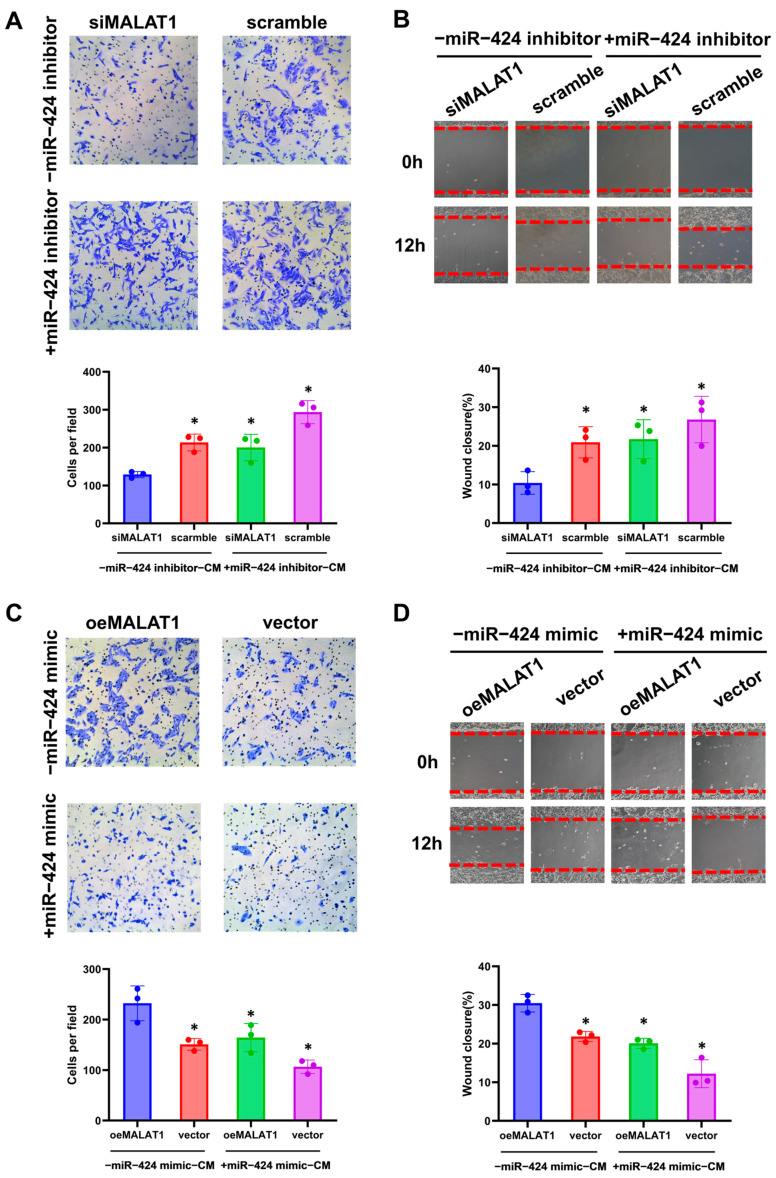
MALAT1 regulates trophoblast invasion and migration through the miR-424/ERRγ/HSD17B1 pathway. (**A**) Representative images of transwell assays after treatment with siMALAT1 and negative controls (scramble), with or without the miR-424 inhibitor, and the quantitative results. * *p* < 0.05 vs. the siMALAT1 group. (**B**) Representative images of wound healing assays after treatment with siMALAT1 and negative controls (scramble), with or without the miR-424 inhibitor, and the quantitative results. * *p* < 0.05 vs. the siMALAT1 group. (**C**) Representative images of transwell assays after treatment with oeMALAT1 and negative controls (vector), with or without the miR-424 mimic, and the quantitative results. * *p* < 0.05 vs. the oeMALAT1 group. (**D**) Representative images of wound healing assays after treatment with oeMALAT1 and negative controls (vector), with or without the miR-424 mimic, and the quantitative results. * *p* < 0.05 vs. the oeMALAT1 group. Statistical data were measurement data and described as the mean ± standard deviation. Original images can be found in the Supplementary Materials.

We meanwhile tested the effect of MALAT1 regulating miR-424 on trophoblast biological functions. Compared with negative-control-transfected cells, trophoblasts transfected with siMALAT1 exhibited a significant decrease in the invasive and migrative ability, as analyzed via transwell and wound healing assays, but the miR-424 inhibitor reversed these effects (Figure 6A,B). In contrast, overexpressing MALAT1 markedly promoted the invasive and migrative capabilities of HTR-8/SVneo cells, while co-transfection with the miR-424 mimic impaired trophoblast functions (Figure 6C,D).

In general, we found that MALAT1 can regulate the paracrine effects of trophoblasts on HUVEC angiogenesis and proliferation, as well as trophoblast invasion and migration, by regulating the miR-424/ERRγ/HSD17B1 pathway, which may contribute to the impaired angiogenesis and placental insufficiency in the placental share of the smaller sFGR fetuses.

### 3.6. MALAT1 Affects the Proliferation and Apoptosis of Trophoblasts under Hypoxia by Sponging miR-424

Impaired angiogenesis and placental insufficiency may lead to hypoxia, which is consistent with previous studies that the growth-restricted twin of sFGR is in a hypoxic environment [22]. As other studies showed that the altered expression of MALAT1 under different oxygen concentrations could affect cell survival under hypoxia [23,24], we further explored the changes in MALAT1 expression in trophoblasts under hypoxia and its effects on trophoblast proliferation and apoptosis.

**Figure 7:**
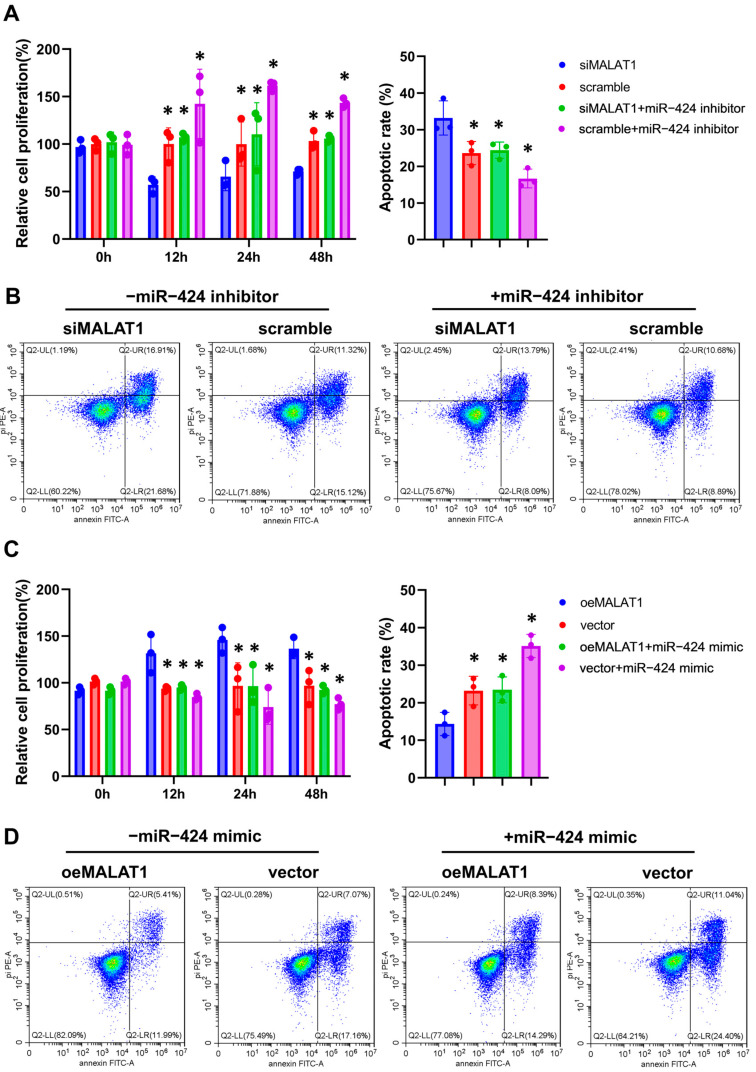
MALAT1/miR-424 affects the proliferation and apoptosis of trophoblasts under hypoxia. (**A**) The ability of trophoblast proliferation after treatment with siMALAT1 and negative controls (scramble), with or without the miR-424 inhibitor, determined via CCK8 assays at different time points of hypoxic culture, and quantitative analysis of (**B**). Relative to negative control (scramble) group, * *p* < 0.05 vs. the siMALAT1 group. (**B**) Representative results of trophoblast apoptosis after treatment with siMALAT1 and negative controls (scramble), with or without the miR-424 inhibitor, cultured for 48 h under hypoxia detected via flow cytometry. (**C**) The ability of trophoblast proliferation after treatment with oeMALAT1 and negative controls (vector), with or without the miR-424 mimic, determined via CCK8 assays at different time points of hypoxic culture, and quantitative analysis of (**D**). Relative to negative control (vector) group, * *p* < 0.05 vs. the oeMALAT1 group. (**D**) Representative results of trophoblast apoptosis after treatment with oeMALAT1 and negative controls (vector), with or without the miR-424 mimic, cultured for 48 h under hypoxia, detected via flow cytometry. Statistical data were measurement data and described as the mean ± standard deviation. Original images can be found in the Supplementary Materials.

The results of CCK8 showed that the proliferation ability of trophoblasts did not decrease after 12 h of hypoxia compared with that under normoxia, but continuous hypoxia after that significantly decreased cell proliferation (Supplementary Figure S3A). After 12, 24, and 48 h of hypoxia culture, MALAT1 expression was significantly upregulated under hypoxia compared with that of cells cultured under normoxia (Supplementary Figure S3B). Further, CCK8 assays and flow cytometry were used to detect the proliferation and apoptosis of trophoblast cells under hypoxia after knockdown and overexpression of MALAT1. Compared with negative control-transfected cells, trophoblasts transfected with siMALAT1 exhibited a significant decrease in growth and a significant increase in apoptosis, but the miR-424 inhibitor reversed these effects (Figure 7A,B). The proliferation capacity of trophoblasts increased and apoptosis decreased in the MALAT1-overexpression group, while co-transfection with the miR-424 mimic impaired trophoblast survival (Figure 7C,D).

## 4. Discussion

**Figure 8:**
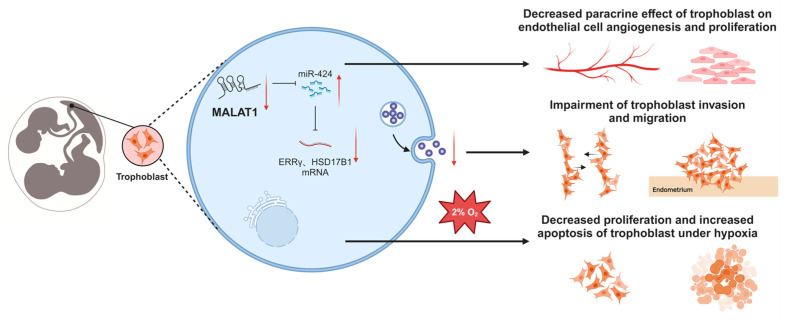
The schematic diagram depicts the role of MALAT1 in regulating the miR-424/ERRγ/HSD17B1 axis in sFGR. The downregulation of MALAT1 in the placental shares of the smaller twins can reduce the expression of ERRγ and HSD17B1 by competitively binding to miR-424, impairing the paracrine effect and biological functions of trophoblasts and the ability of trophoblasts to compensate for hypoxia, which may be involved in the pathogenesis of sFGR through various aspects.

MC twins are derived from one fertilized zygote, with the same intrauterine environment and almost identical genetic materials, so epigenetic regulatory mechanisms may play an important role in the pathogenesis of sFGR. As a part of epigenetic studies, lncRNA has attracted attention in recent years for its role in various physiological and pathological processes, including placenta and fetal development [13,25]. In the present study, we found that a lncRNA, MALAT1, was significantly decreased in the placental share of the smaller twin in sFGR, inhibiting the angiogenic paracrine effects of trophoblasts through the miR-424/ERRγ/HSD17B1 axis, and thus the tube formation and proliferation ability of the endothelium was hampered. Moreover, decreased MALAT1 impeded trophoblast migration and invasion and cell survival under hypoxia (Figure 8). These results suggest that decreased MALAT1 in the placental region of the smaller twin may lead to impaired angiogenesis and a lower placental share in the growth-restricted twin, which may be involved in the pathogenesis of sFGR from multiple perspectives.

MALAT1 was initially found to be significantly elevated in metastatic lung cancer cells [26], and studies have shown that it plays an important role in the angiogenic capacity, invasion, and apoptosis of tumor cells, which is a potential biomarker for tumor prediction and treatment [14]. Due to the similarity of functional and molecular pathways between placental trophoblast and tumor cells [27], MALAT1 has also been studied in pregnancy-related diseases, but its role in regulating placental angiogenesis is under-elucidated. As a highly vascularized organ, placenta perfusion plays an important role in the adequate nutritional support and metabolite clearance of the fetus, so impaired placental angiogenesis leading to placenta malperfusion may result in intrauterine fetal distress, preeclampsia, and other pregnancy complications [28,29]. Recent studies on the placental vascular network using CT angiography, 3D energy Doppler ultrasound, and other technologies [30,31] have shown that the placental volume and blood perfusion of the smaller twin in sFGR are poorer than the larger twin, and the possible mechanism is under investigation. It has been suggested that trophoblasts may be the main source of angiogenic regulators, such as vascular endothelial growth factor (VEGF), placental growth factor (PIGF), angiopoietin (ANGPT), etc., to regulate the chemotaxis, migration, tubular formation, and vascular stability of endothelial cells and thus the normal development of the placental vascular network [32,33,34]. The results of immunohistochemical staining of placental blood vessels in our study also showed that the number of blood vessels and the volume density in the placental region of the smaller twin in sFGR were significantly reduced, and further in vitro experiments showed that the decrease in MALAT1 in trophoblasts impaired its paracrine effect on the tube formation and proliferation of endothelial cells through miR-424. Qiu Jun-Jun et al. showed that the significant increase in MALAT1 in metastatic epithelial ovarian cancer cells promotes angiogenesis by activating the expression of VEGF, PIGF, and other factors [35], while Teng Fei et al. showed that the decrease in miR-424 in liver cancer cells promotes angiogenesis by increasing the expression of another protein, E2F7 [36]. Our results showed that MALAT1 regulates HSD17B1 expression through miR-424, and HSD17B1 is a key enzyme rich in trophoblasts for the production of estradiol, which can induce the expression of VEGF and increase the vascularized area in the placenta [37]. Therefore, our study suggested that a decrease in MALAT1 may be related to impaired angiogenesis in sFGR fetuses by attenuating the paracrine hormones of trophoblasts through the miR-424/ERRγ/HSD17B1 pathway, which needs further validation.

In addition to the importance of placental vascular development, it is considered that the impaired function of the trophoblast layer is also one of the leading causes of abnormal fetal growth. Our results showed that decreased MALAT1 expression weakened the proliferation, invasion, and migration ability of trophoblasts, which was similar with studies in preeclampsia and recurrent spontaneous abortion [16,17,38]. FISH results showed that MALAT1 was mainly located in the trophoblast cytoplasm, which was consistent with Li Qi et al.’s research [16]. Subsequent results first demonstrated that MALAT1 can regulate the expression of ERRγ and HSD17B1 in trophoblasts through the competing endogenous RNA (ceRNA) mechanism as a molecular sponge of miR-424 and regulate trophoblast functions through the miR-424/ERRγ/HSD17B1 axis. MALAT1 has also been found to be a ceRNA of other miRNAs in trophoblasts [17,39], but as a cytoplasm-located lncRNA, it may regulate cell functions via additional mechanisms, such as affecting gene transcription, binding to proteins, influencing protein post-translational modification or localization, etc. [40], which can be further studied in trophoblasts.

Moreover, studies have shown that hypoxia-induced trophoblast damage is significantly increased in the placenta region of the smaller twin in sFGR, which may further aggravate cell dysfunction and placental dysplasia [41]. However, other studies have suggested that moderate or short hypoxia can activate cell metabolism, proliferation, and other pathways, which is beneficial for cell adaptation, such as the low apoptosis rate of trophoblasts under hypoxia in early pregnancy [42]. The results of our study showed that the survival of trophoblasts did not decrease under a short period of hypoxia, but cell proliferation was significantly impaired and apoptosis was significantly increased under continuous hypoxia, suggesting that trophoblasts could compensate to a certain extent under hypoxia. At the same time, we detected that MALAT1 was consistently increased in trophoblasts under hypoxia compared with that under normoxia culture, which was similar to that in various cell types under hypoxia [43,44,45]. Studies have shown that an increase in MALAT1 under hypoxia can alleviate cell damage in ischemic brain injury and enhance cell survival in endometriosis [23,46], while MALAT1 may exacerbate hypoxic-reoxygenation damage in liver cells or hypoxia-induced lung dysfunction [24,47]. So far, no studies have been conducted to investigate the effect of MALAT1 on trophoblasts under hypoxia. In order to explore whether MALAT1 affects trophoblast survival under hypoxia and its mechanism, we investigated it from two aspects, cell proliferation and apoptosis. Our results showed that the proliferation of trophoblasts decreased and apoptosis increased significantly after MALAT1 knockdown, while the proliferation increased and apoptosis decreased in the MALAT1-overexpression group, suggesting that the increased expression of MALAT1 in trophoblasts under hypoxia may be a compensatory response against stress by decreasing apoptosis and promoting proliferation. Additionally, we observed that the expression of MALAT1, ERRγ, and HSD17B1 was increased in the placental shares of the smaller twins in the control group but not significantly (*p* > 0.05), which may contribute to the maintenance of placental functions supporting normal fetal growth. In contrast, decreased MALAT1 in the placental region of sFGR may interrupt the compensatory response of trophoblasts under hypoxia and further aggravate trophoblast damage, thus promoting the inconsistent growth between twins.

Previous studies have suggested that miRNAs [9,41], such as miR-210 and miR-199a, are involved in the occurrence and development of sFGR by targeting genes related to cell proliferation and oxidative stress. Our results showed that another miRNA, miR-424, was also closely related to sFGR, as it was significantly increased in the placental shares of the smaller twins and impaired trophoblast functions, which is consistent with our previous research findings in FGR [10]. Previous studies have shown that ERRγ and HSD17B1 can regulate hormone synthesis, energy metabolism, and other processes, playing an important role in trophoblast differentiation and placental development, and their expression was decreased in FGR and preeclampsia [48,49]. The results of this study showed that the expression of ERRγ and HSD17B1 was also reduced in the corresponding placental region of the smaller twin in sFGR, impairing the paracrine and biological functions of trophoblasts. Therefore, our present study provides more evidence that the miR-424/ERRγ/HSD17B1 axis is closely related to placental development and fetal growth.

However, there are still some limitations in our study. Firstly, the number of clinical cases for uncovering the differential expression of corresponding molecules is relatively small, and specimen collection of appropriate cases is ongoing to expand the sample size in the future. Secondly, the specific pro-angiogenic paracrine molecules of trophoblasts regulated by MALAT1 that may be associated with sFGR need to be further explored. Lastly, though our present results preliminarily showed the relationship between MALAT1 and fetal growth, in the future, it is necessary to detect the expression level in other pregnancies with fetal growth restriction and to conduct animal experiments to further verify the specific role of MALAT1 in regulating placental angiogenesis, the comprehensive function of placenta, and fetal growth.

## 5. Conclusions

In conclusion, our study revealed the involvement of the MALAT1/miR-424/ERRγ/HSD17B1 axis in sFGR. Our novel findings showed that decreased MALAT1 would reduce ERRγ and HSD17B1 in trophoblasts by binding to miR-424, thus impairing the paracrine effect of trophoblasts on endothelial cell angiogenesis, the biological functions of trophoblasts, and the ability of trophoblasts to compensate under hypoxia, underlying the role of the MALAT1 decline in the pathogenesis of sFGR through multiple aspects. The present study indicates that MALAT1 plays important regulatory roles in placenta functions and fetal development and provides evidence of the epigenetic mechanism through which MALAT1 may be associated with phenotypic discrepancies in monozygotic twins.

## Figures and Tables

**Table 1:** The primer sequences for RT-qPCR.

Gene	Forward (5′-3′)	Reverse (5′-3′)
MALAT1	GGTCTCCCCACAAGCAACTT	AACCCACCAAAGACCTCGAC
ERRγ	GTTCAGCCAGCCAAAAAGCC	AACCCACCAAAGACCTCGAC
HSD17B1	AGCTGGACGTAAGGGACTCA	ATGCTTCGCCCATCCAATGA
β-actin	CATGTACGTTGCTATCCAGGC	CTCCTTAATGTCACGCACGAT
miR-424	AACAAGCAGCAGCAATTCATGTTTT	Provided by the kit EZB-miRT2

**Table 2:** The clinical characteristics of participants whose placental tissues were studied in the present study.

Characteristic	sFGR (n = 8)	Control (n = 8)	*p* Value
Age (years)	28 (27–32)	35 (32–38)	0.007
Gestational age at delivery (weeks)	34 + 1 (32 + 1–35 + 5)	36 + 1 (35 + 6–36 + 6)	0.005
Birth weight (g)			
Larger twin	2140 (1870–2320)	2420 (2360–2580)	0.021
Smaller twin	1480 (1230–1670)	2270 (2160–2540)	<0.001
Birth weight discordance (%)	32.9 (28.4–35.0)	5.1 (1.8–8.7)	<0.001
Placenta share of smaller twins (%)	22.2 (18.5–28.6)	41.7 (40.0–47.6)	<0.001

Data are expressed as the median (interquartile range) or n (%).

## Data Availability

The original contributions presented in the study are included in the article and supplementary materials, and further inquiries can be directed to the corresponding author.

## Supplementary Materials

The following supporting information can be downloaded at: https://www.mdpi.com/article/10.3390/biom14080988/s1, Figure S1: Expression of MALAT1, miR-424, ERRγ, and HSD17B1 in placenta of sFGR group and control group. Figure S2: MALAT1 affects expression of ERRγ and HSD17B1. Figure S3: The proliferation and MALAT1 expression in trophoblasts are regulated under hypoxia. Original western blot and microscopy images can be found in the Supplementary Materials.
