# Supplementary material for: The Role of MALAT1 in Regulating the Proangiogenic Functions, Invasion, and Migration of Trophoblasts in Selective Fetal Growth Restriction
Source: Biomolecules. 2024 Aug 11;14(8):988. doi: 10.3390/biom14080988 (PMC11352967; doi:10.3390/biom14080988)
Supplement: Supplementary file 1 [file biomolecules-14-00988-s001.zip › biomolecules-3111426-supplementary.pdf]

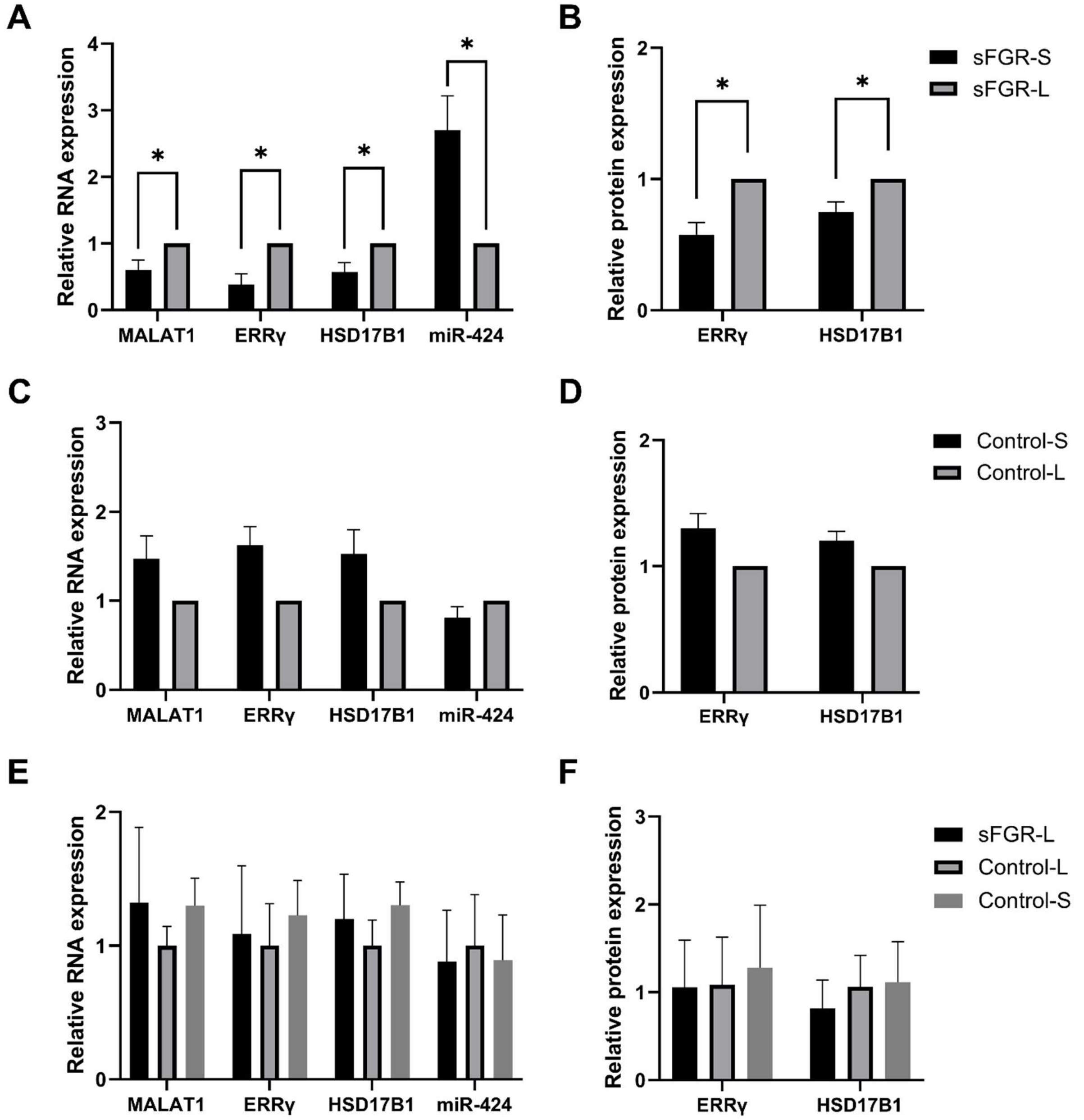


**Supplementary Figure S1.** Expression of MALAT1, miR-424, ERRγ and HSD17B1 in placenta of sFGR group and control group. (A) RNA expression levels of corresponding molecules in sFGR group. Relative to the larger co-twin in each pair; **P* < 0.05 vs larger fetuses. (B) The quantitative results of protein expression levels of ERRγ and HSD17B1 in sFGR group. Relative to the larger co-twin in each pair; **P* < 0.05 vs larger fetuses. (C) RNA expression levels of corresponding molecules in larger fetuses of sFGR group and control twins. Relative to larger fetuses of control group. (D) The quantitative results of protein expression levels of ERRγ and HSD17B1 in larger fetuses of sFGR group and control twins. Relative to larger fetuses of control group. Statistical data were measurement data and described as mean ± standard deviation. The paired-sample t test was used for comparison between smaller fetus and the larger co-twin, and the one-way analysis of variance was used for comparison among larger fetuses of sFGR group and control twins, followed by the Tukey's post hoc test. L, larger twins; S, smaller twins

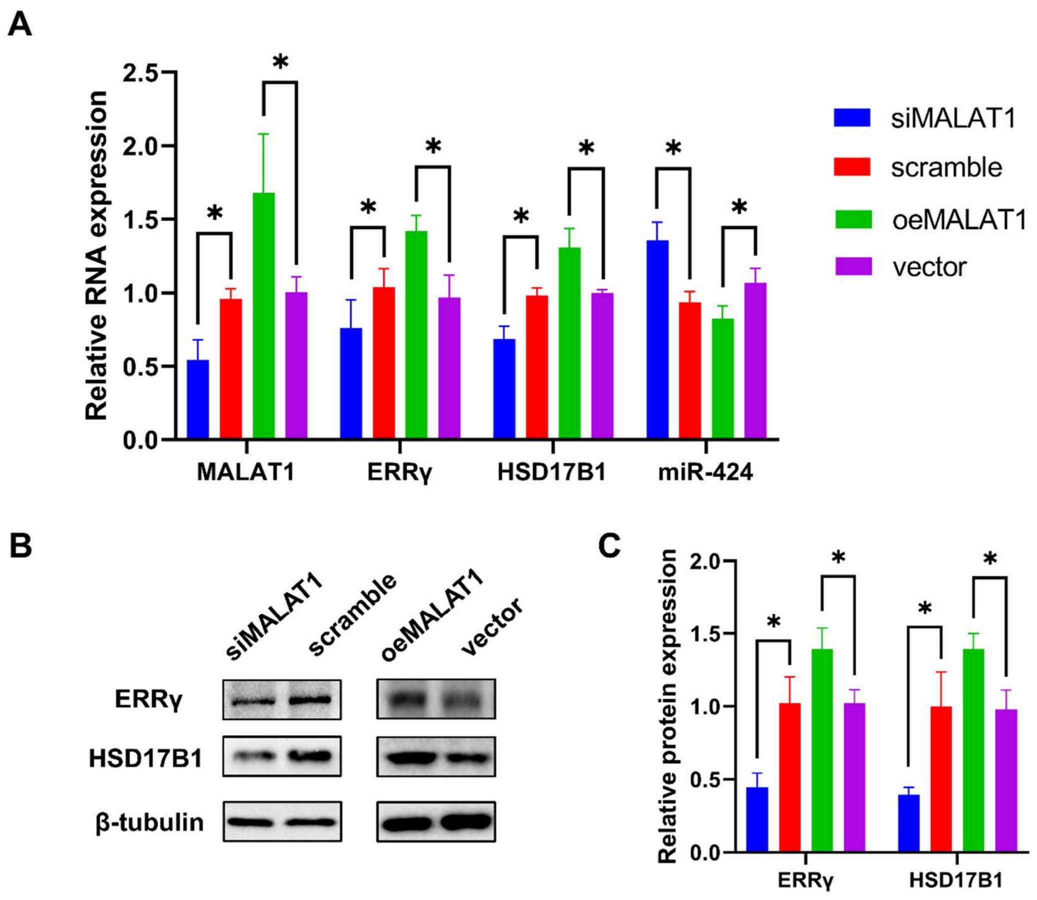


**Supplementary Figure S2.** MALAT1 affects expression of ERRγ and HSD17B1. (A) RNA expression levels of miR-424, ERRγ and HSD17B1 in response to the treatment of siMALAT1, oeMALAT1 and their corresponding controls determined by RT-qPCR. **P* < 0.05 vs corresponding controls. (B) The Western blot results of ERRγ and HSD17B1 in response to the treatment of siMALAT1, oeMALAT1 and their corresponding controls. (C) The quantitative analysis of Western blot results of ERRγ and HSD17B1. **P* < 0.05 vs corresponding controls. Statistical data were measurement data and described as mean ± standard deviation. The experiments were repeated three times independently.

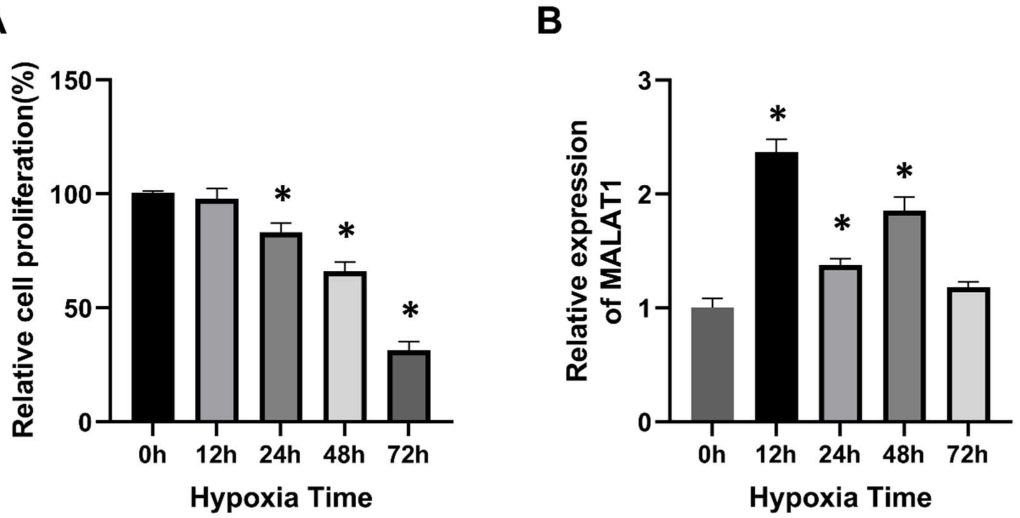


**Supplementary Figure S3.** The proliferation and MALAT1 expression of trophoblast are regulated under hypoxia. (A) The ability of trophoblast proliferation after different periods of culture under hypoxia determined by CCK8 assays. Relative to cells cultured under normoxia after different periods of time. (B) The expression of MALAT1 after different periods of culture under hypoxia determined by RT-qPCR. Relative to cells cultured under normoxia after different periods of time. Statistical data were measurement data and described as mean ± standard deviation. The experiments were repeated three times independently.
